# Supplementary material for: Detect coastal disturbances and climate change effects in coralligenous community through sentinel stations
Source: PLoS One. 2020 May 5;15(5):e0231641. doi: 10.1371/journal.pone.0231641 (PMC7200002; doi:10.1371/journal.pone.0231641)
Supplement: Attachment — Submitted filename: PONE-D-19-25987_reviewer PEIRANO.docx [file pone.0231641.s002.docx]

## PLOS ONE

### Long-term pattern of sessile rocky bottom bioindicators. Testing early-warning system for detecting coastal disturbances and climate change effects

--Manuscript Draft--

| **Manuscript Number:** | PONE-D-19-25987 |
| --- | --- |
| **Article Type:** | Research Article |
| **Full Title:** | Long-term pattern of sessile rocky bottom bioindicators. Testing early-warning system for detecting coastal disturbances and climate change effects |
| **Short Title:** | Long-term pattern of sessile rocky bottom. Testing early-warning system for detecting coastal disturbances |
| **Corresponding Author:** | Manuel Maestre  Universidad de Sevilla Facultad de Biologia Sevilla, SPAIN |
| **Keywords:** | Long-term monitoring; Indicator species; Coralligenous; Rapid assessment; MPAs management |
| **Abstract:** | This study was implemented to validate the Sessile Bioindicators in Permanent Quadrats (SBPQ) underwater environmental alert method. The SBPQ focuses on the monitoring of preselected sessile and sensitive benthic species associated with rocky coralligenous habitats using permanent quadrats in underwater sentinel stations. The selected target species have been well documented as bioindicators that disappear when environmental conditions deteriorate. However, whether these species are good indicators of stability or, in contrast, suffer variations in long-term coverage has not been verified. The purpose of this study was to confirm the untested part of the method based on the hypothesis that, over a long temporal series in a highly structured and biodiverse coralligenous assemblage, the cover of sensitive sessile species does not change over time if the environmental stability characterising the habitat is not altered. Over a ten-year period (2005-2014), the sublittoral sessile biota in the Straits of Gibraltar Natural Park on the southern Iberian Peninsula was monitored at a 28 m- deep underwater sentinel station. Analyses of the coverages of target indicator species ( i.e. , Paramuricea clavata and Astroides calycularis ) together with other accompanying sessile organisms based on the periodic superimposition of gridded images from horizontal and vertical rocky surfaces allowed us to assess the effectiveness of the method. We conclude that no alterations occurred during the study period; only minimal fluctuations in cover were detected, and the method is reliable for detecting biological changes in ecosystems found in other geographical areas containing the chosen indicator species at similar dominance levels.  The SBPQ is a non-invasive and low-cost protocol; it uses sessile target species (indicators) to detect environmental alterations (natural or anthropic) at either the local or global ( i.e. , climate change) scale and the intrusion of invasive species. |
| **Order of Authors:** | José Carlos García-Gómez |
|  | Alexandre Roi González |
|  | Manuel Jesús Maestre |
|  | Free Espinosa |
| **Additional Information:** |  |
| **Question** | **Response** |
| **Financial Disclosure**  Enter a financial disclosure statement that describes the sources of funding for the work included in this submission. Review the submission guidelines for detailed | The results presented here were supported by EU-funded projects (Feder, Interreg IIIc, and MedPAN) and the Ministry of the Environment and Spatial Planning (CMAyOT) of the Regional Government of Andalusia. The funders had no role in study design, data collection and analysis, decision to publish, or preparation of the manuscript. |

| requirements. View published research articles from *PLOS ONE* for specific examples.  This statement is required for submission and **will appear in the published article** if the submission is accepted. Please make sure it is accurate. |  |
| --- | --- |
| **Unfunded studies**  Enter: *The author(s) received no specific funding for this work.*  **Funded studies**  Enter a statement with the following details:   - Initials of the authors who received each award - Grant numbers awarded to each author - The full name of each funder - URL of each funder website - Did the sponsors or funders play any role in the study design, data collection and analysis, decision to publish, or preparation of the manuscript? - **NO** - Include this sentence at the end of your statement: *The funders had no role in study design, data collection and analysis, decision to publish, or preparation of the manuscript.* - **YES** - Specify the role(s) played. |  |
| * typeset |  |
| **Competing Interests**  Use the instructions below to enter a competing interest statement for this submission. On behalf of all authors, disclose any competing interests that could be perceived to bias this work—acknowledging all financial support and any other relevant financial or non- financial competing interests.  This statement **will appear in the published article** if the submission is accepted. Please make sure it is  accurate. View published research articles from *PLOS ONE* for specific examples. | The authors have declared that no competing interests exist. |

| **NO authors have competing interests**  Enter: *The authors have declared that no competing interests exist.*  **Authors with competing interests**  Enter competing interest details beginning with this statement:  *I have read the journal's policy and the authors of this manuscript have the following competing interests: [insert competing interests here]* |  |
| --- | --- |
| * typeset |  |
| **Ethics Statement**  Enter an ethics statement for this submission. This statement is required if the study involved:   - Human participants - Human specimens or tissue - Vertebrate animals or cephalopods - Vertebrate embryos or tissues - Field research   Write "N/A" if the submission does not require an ethics statement.  General guidance is provided below. Consult the submission guidelines for detailed instructions. **Make sure that all information entered here is included in the Methods section of the manuscript.** | N/A |

| **Format for specific study types**  **Human Subject Research (involving human participants and/or tissue)**   - Give the name of the institutional review board or ethics committee that approved the study - Include the approval number and/or a statement indicating approval of this research - Indicate the form of consent obtained (written/oral) or the reason that consent was not obtained (e.g. the data were analyzed anonymously)   **Animal Research (involving vertebrate animals, embryos or tissues)**   - Provide the name of the Institutional Animal   Care and Use Committee (IACUC) or other relevant ethics board that reviewed the study protocol, and indicate whether they approved this research or granted a formal waiver of ethical approval   - Include an approval number if one was obtained - If the study involved *non-human primates*, add *additional details* about animal welfare and steps taken to ameliorate suffering - If anesthesia, euthanasia, or any kind of animal sacrifice is part of the study, include briefly which substances and/or methods were applied   **Field Research**  Include the following details if this study involves the collection of plant, animal, or other materials from a natural setting:   - Field permit number - Name of the institution or relevant body that granted permission | |  |
| --- | --- | --- |
|  | |  |
| **Data Availability**  Authors are required to make all data underlying the findings described fully available, without restriction, and from the time of publication. PLOS allows rare exceptions to address legal and ethical concerns. See the PLOS Data Policy and FAQ for detailed information. | | Yes - all data are fully available without restriction |
|  |  |  |

| A Data Availability Statement describing where the data can be found is required at submission. Your answers to this question constitute the Data Availability Statement and **will be published in the article**, if accepted. |  |
| --- | --- |
| **Important:** Stating ‘data available on request from the author’ is not sufficient. If your data are only available upon request, select ‘No’ for the first question and explain your exceptional situation in the text box. |  |
| Do the authors confirm that all data underlying the findings described in their manuscript are fully available without restriction? |  |
| **Describe where the data may be found in full sentences. If you are copying our sample text, replace any instances of XXX with the appropriate details.**   - If the data are **held or will be held in a public repository**, include URLs, accession numbers or DOIs. If this information will only be available after acceptance, indicate this by ticking the box below. For example: *All XXX files are available from the XXX database (accession number(s) XXX, XXX.*). - If the data are all contained **within the manuscript and/or Supporting Information files**, enter the following: *All relevant data are within the manuscript and its Supporting Information files.* - If neither of these applies but you are able to provide **details of access elsewhere**, with or without limitations, please do so. For example:   *Data cannot be shared publicly because of [XXX]. Data are available from the XXX Institutional Data Access / Ethics Committee (contact via XXX) for researchers who meet the criteria for access to confidential data.*  *The data underlying the results presented in the study are available from (include the name of the third party* | All relevant data are within the manuscript and its Supporting Information files. |

| *and contact information or URL).*   - This text is appropriate if the data are owned by a third party and authors do not have permission to share the data.   * typeset |  |
| --- | --- |
| Additional data availability information: |  |

Manuscript	Click here to download Manuscript renamed_3b1d7.docx

1

### 2 Long-term pattern of sessile rocky bottom bioindicators. Testing early-

3 **warning system for detecting coastal disturbances and climate change**
4 **effects**

5

#### 6 José C. García-Gómez1*¶, Alexandre R. González1¶, Manuel J. Maestre1¶, Free

7 **Espinosa1¶**

8

9 1 Laboratory of Marine Biology, Department of Zoology, Faculty of Biology, University
10 of Seville, Seville, Spain 11
12 ^*^Corresponding author
13 E-mail: [jcgarcia@us.es](mailto:jcgarcia@us.es) (JCGG) 14

15	¶These authors contributed equally to this work 16

17

18

19

20

21

# 23 Abstract

24 This study was implemented to validate the Sessile Bioindicators in Permanent
25 Quadrats (SBPQ) underwater environmental alert method.
26 The SBPQ is a non-invasive and low-cost protocol; it uses sessile target species
27 (indicators) to detect environmental alterations (natural or anthropic) at either the local or
28 global (*i.e.*, climate change) scale and the intrusion of invasive species.

The SBPQ focuses on the

30 monitoring of preselected sessile and sensitive benthic species associated with rocky
31 coralligenous habitats using permanent quadrats in sentinel stations. The
32 selected target species have been well documented as bioindicators that disappear when
33 environmental conditions deteriorate. However, whether these species are good indicators
34 of stability or, in contrast, suffer variations in long-term coverage has not been verified.
35 The purpose of this study was to confirm the untested part of the method based on the
36 hypothesis that, over a long temporal series in a highly structured and biodiverse
37 coralligenous assemblage, the cover of sensitive sessile species does not change over time
38 if the environmental stability characterising the habitat is not altered. Over a ten-year
39 period (2005-2014), the sublittoral sessile biota in the Straits of Gibraltar Natural Park on
40 the southern Iberian Peninsula was monitored at a 28 m-deep underwater sentinel station.
41 Analyses of the coverages of target indicator species (*i.e.*, *Paramuricea clavata* and
42 *Astroides calycularis*) together with other accompanying sessile organisms based on the
43 periodic superimposition of gridded images from horizontal and vertical rocky surfaces
44 allowed us to assess the effectiveness of the method. We conclude that no alterations
45 occurred during the study period; only minimal fluctuations in cover were detected, and
46 the method is reliable for detecting biological changes in ecosystems found in other
47 geographical areas containing the chosen indicator species at similar dominance levels.

# 48 Introduction

49 The long-term evaluation and quantification of changes in species are crucial for
50 our knowledge of various marine ecosystems [1, 2] and constitute useful tools for the
51 environmental monitoring, management, and conservation of coastal zones and their
52 associated marine protected areas.
54 Historically, methodological difficulties related to the monitoring and study of
55 subtidal communities have led to their neglect in many environmental monitoring
56 programs [3], particularly in relation to rocky habitats because of their wide
57 heterogeneities, the fixed nature of the resident sessile organisms, the frequent difficulty
58 of achieving access, and the fact that the sampling must be undertaken through indirect
59 methods from boats. Warwick [4] indicated that the study of these communities on wide
60 spatial scales involves a series of technical and implementation difficulties that are not
61 present for work on soft bottoms, the water column, or the intertidal zone. For these
62 reasons, studies related to the environmental monitoring, surveillance, and impacts of
63 various factors on the sublittoral benthic environment have focused on endofauna
64 (macroinvertebrates) associated with soft substrates [5-12]. Many biotic indices based on
65 these animals have been proposed [13-14], and many of these indices have been used for
66 or adapted to the requirements of the European Community Water Framework Directive

67	[15-17].

69 Indeed, regarding monitoring methods based on indicators in the scope of the
70 European Community Directives [18-19], the research has centred on macroalgae on hard
71 substrates [20-27] and seagrasses [28-30], whereas macroinvertebrates associated with
72 high-diversity rocky subtidal habitats (which have greater abundances of sensitive
73 indicator species) have been less well studied. However, some studies have sought to
74 establish the ecological statuses of coralligenous assemblages [31-37]. The environmental
75 information that can be provided by these assemblages is very powerful and reliable [38-
76 41]; therefore, this faunal approach to rocky habitats could help to fill an important
77 knowledge gap within the European Community Directives [18-19]. 78
79 Of particular importance is the information that can be provided by pre-
80 coralligenous and coralligenous rocky bottoms because of their high biodiversities and
81 the abundances of sensitive colonial species with long life cycles. However, there are very
82 few long-term studies of species that are representative of coralligenous communities [42-
83 43], although short- and medium-term studies are more common [44-45]. 84
85 In contrast, growing concern about the effects of climate change has led to more
86 studies of epibenthic (rocky bottom) species in relation to coral bleaching [46-48],
87 seagrass meadows [49], invasive alien species [50-51], and even the role of the marine
88 reserve biota in climate change monitoring [52-54]. These studies have also highlighted
89 the severe environmental impacts on benthic organisms that have arisen from abnormal
90 temperature increase events in the western Mediterranean [55-57]. 91
92 The great fragility of highly structured and mature benthic communities associated
93 with hard substrates, such as those of pre-coralligenous and coralligenous areas, has
94 encouraged non-invasive study methods based on video footage or photos of species that
95 are fixed to the substrate in random quadrats [34, 45, 58-65]. However, the absence of
96 long-term series that have empirically validated these methodologies as systems for
97 detecting changes in communities has been limiting.

98

99	Additionally, previous studies have focused on characterising and/or monitoring

100

benthic communities, which implies greater complexity with respect to implementation

101

mainly due to the high diversity of species present in these enclaves, which, in turn, entails

102

greater effort and difficulty associated with the identification of the taxa. However, the

103

Sessile Bioindicators in Permanent Quadrats (SBPQ) methodology, which was proposed

104

by one of the authors of the present paper [41], and the present study aims to validate,

105

involves an approach that differs from those previously mentioned. The SBPQ method

106

uses permanent quadrats and does not focus on monitoring the benthic community of the

107

hard substrate; rather, it focuses on monitoring the evolution of the coverage by sessile

108

target species (*i.e.*, sensitive species with medium or long life cycles) on hard substrates

109

in quadrats that are selectively and permanently installed at “SBPQ sentinel stations”.

110

This method provides an early-warning system for any possible significant change that

111

could be related to local, regional, or global (*i.e.*, climate change) environmental

112

113

alterations and aids the early detection of exotic species in the benthic macrobiota.

114

## Objectives

115

This study aims to validate the underwater environmental SBPQ alert method,

116

which focuses on monitoring preselected sessile and sensitive benthic species associated

117

with rocky coralligenous habitats using permanent quadrats in underwater sentinel

118

stations. The selected target species have been well documented as bioindicators that

119

disappear when environmental conditions deteriorate. However, whether these species

120

are good indicators of stability, or, in contrast, if they exhibit variations in long-term

121

coverage, has not been verified. The purpose of the present study was to confirm the

122

untested portion of the method based on the hypothesis that, over a long temporal series

123

(a ten-year period in this study) in a highly structured and biodiverse coralligenous

124

assemblage, the cover of sensitive sessile species (*i.e.*, the target species) does not change

125

over time if the environmental stability of the habitat is not altered. This lack of change

126

in coverage in stable environmental conditions would be a key aspect for conferring

127

reliability and robustness on the SBPQ underwater environmental warning system and

128

would thus confirm its usefulness because the system would be sensitive to changes in

129

the coverages of the target species due to physico-chemical changes in the environment,

130

including gradual elevations of temperature that might result from global warming and

131

132

changes due to the introduction of exotic species.

133

The long-term objective of this work is to implement the SBPQ methodology in

134

well-conserved areas of the Spanish coast so that those areas can act as “SBPQ sentinel

135

stations” in the event of possible disturbances. The method has been developed as a

136

simple management tool for use by scientists and specialised technicians in addition to

137

138

139

diving clubs that frequent certain areas.

# Materials and methods

140

## Study zone

141

This study was performed on the benthic community of a sentry station on a rocky

142

bottom at

The station is situated within the Strait of Gibraltar Natural Park (southern Spain)

144

inside the Grade A protection zone (*i.e.*, the maximum protection area within the Natural

145

Park) in the Punta Carnero locality (Algeciras), but it is proximal to zones under strong

146

147

anthropic pressure (Fig 1).

148

149

#### Fig 1. Location of the sampling point in the Straits Natural Park.

150

Despite its enormous ecological wealth and high level of protection, the SBPQ

151

sentry station is at notable risk of anthropogenic disturbance. This is due to both to its

152

proximity to the particularly industrialised Bay of Algeciras [66, 67] and the high level

153

of marine traffic through the area. Due to the second factor, the last decade has seen

154

polluting events related to hydrocarbon spills of varying magnitudes including serious

155

156

spills from the ships *Sierra Nava* and *New Flame*, both of which occurred in 2007 [68].

157

Data regarding the bottom water temperature in the study area were obtained from

158

159

data base of the regional environmental authorities [69].

160

## Application of the SBPQ methodology for its validation: Target

161

## species, permanent grids, and underwater sentinel stations

162

The SBPQ method was proposed by García-Gómez [41] , but its validation is

163

pending a study of a long time series that could confirm that the coverages of long-cycle

164

indicator species do not change over time in undisturbed benthic environments with high

165

levels of biodiversity unless a significant environmental alteration is introduced into and

166

modifies the system. Such environmental alterations would be measurable. The SBPQ

167

method focuses on monitoring the evolution of the coverages of sessile indicator target

168

species (such species should be sensitive and have medium or long life cycles) on hard

169

substrates in quadrats that are selectively and permanently installed at “SBPQ sentinel

170

stations”. This monitoring could provide an early warning system regarding any possible

171

significant changes that could be related to local, regional, or global (*i.e.*, climate change)

172

environmental alterations. The quadrats are periodically monitored after they are

173

selectively set in areas in which the indicator species are frequent or very abundant; thus,

174

the potential regressions of these species would be easily detectable by decreases in their

175

coverages. These species should always be moderate to large (preferably colonial) and

176

highly visible while immersed. Photographs of the quadrats (a non-invasive method)

177

collected at different times allow for comparisons of the coverages via image analysis

178

techniques and a final statistical analysis (see "Statistical analyses"). This analysis will

179

determine whether, after an elapsed time (t), there are significant differences between the

180

coverages of the target species between quadrats and therefore whether the occurrence of

181

182

a potential environment-impacting event can be inferred.

183

## Choice of indicator species

184

The anthozoans *Astroides calycularis* (Pallas, 1766) and *Paramuricea clavata*

185

(Risso, 1826), which are currently listed as 'vulnerable' by the IUCN, were selected for

186

monitoring. These species are vulnerable to increases in temperature and sensitive to

187

deterioration of the environmental quality of the marine environment. Both species are

188

well referenced in ecological studies of coastal benthos [40, 41, 57, 70-76]. Moreover,

189

some studies have indicated that typical coralligenous species, such as *Paramuricea*

190

*clavata,* exhibit extremely low temporal variability [77]. Additionally, these species are

191

highly representative of the community under study. Therefore, the quantitative stability

192

(cover) of these sensitive species over time in the absence of phenotypic signs of stress

193

(*e.g.*, epibiosis, partial necrosis, bleaching) would allow us to infer that the community

194

has remained healthy. Simultaneously, the coverages of other species (*i.e.*, calcareous

195

algae *Mesophyllum* sp., white gorgonia *Eunicella* sp., and sponge *Crambe* sp.) were

196

measured. Previous research indicates that serious perturbances of infralittoral rocky

197

communities are usually reflected in the entire assemblage (or at least in an important

198

number of species) [56, 78]. Therefore, focusing the monitoring effort on sensitive

199

species that are representative of the community should allow the appropriate inferences

200

201

to be drawn.

202

## Sampling procedure

203

Four fixed 1-m^2^ PVC quadrats were installed in two different locations. One setup

204

had a vertical orientation (shady), and the other had a horizontal orientation (sunny) at a depth of 28 m (coordinates 36º 4,770 N - 05º 25,184 W; GPS, DATUM WGS

143

84)..

205

The quadrats were placed on surfaces containing

206

207

target species The covers were monitored over ten consecutive years (2005-2014)

212

All of the quadrats were filmed, and photos were captured from each of the videos.

213

To minimise any possible problems due to the lower resolution of images captured from

214

video footage with respect to digital photos, 50x50-cm areas were sequentially filmed

215

rather than filming the entire 1x1-m quadrats. Adding these filming units together to

216

achieve a sample size that was comparable to that used when directly counting quadrats

217

is a valid option for reconciling the resolution of the image with the area of cover [63, 79-

218

219

80].

220

The obtained photos were digitally manipulated using the *Adobe Photoshop* 6.0

221

(2000) program as follows: four 50x50-cm areas were created for each quadrat (Fig 2A);

222

the contrast and colour saturation were adjusted (Fig 2B); a complete digital frame was

223

created (Fig 2C); and a digital grid that was adjusted to the perimeter of the monitoring

224

area was superimposed (Fig 2D). I to correct the angular deformation

229

230

#### Fig 2. Digital manipulation of the captured images prior to analysis.

231

To assess the degree of the cover of the target species, a system involving the

232

determination of the presence/absence of cover using the digital grid was applied. This

233

system aided estimation accuracy [81, 82] and optimised the working time [58, 80, 83].

234

Nevertheless, it should be noted that, in multistratified communities (as was the case with

235

the clearly dominant macroalgae), the system tends to underestimate the cover [58, 63,

236

81-84] because larger species “mask” those that developed underneath them. In the case

237

of the species and communities specifically selected in this study, this masking

238

phenomenon was not significant due to the morphological characteristics of the

239

240

organisms.

241

All of the underwater filming tasks and the later digital treatments, interpretation,

242

and analyses of the images in the laboratory were performed by the same researcher to

243

avoid any potential problems due to divergence between different observers [85]. A total

244

245

of 320 photographs were analysed.

246

## Statistical analyses

247

Repeated measures analysis of variance (RM-ANOVA) was applied to test

248

whether the mean coverage of the target species significantly varied either through time

249

(intra-subject factor ‘time’; ten levels: 2005 to 2014) or according to orientation (inter-

250

subject factor ‘orientation’; two levels: horizontal and vertical). Mauchly’s test of

251

sphericity [86] was used to test the assumption that the variances in the differences

252

between all possible pairs of groups were equal. When the sphericity condition was not

253

verified, the F test value was corrected with the Greenhouse-Geisser epsilon index [87,

254

88]. The factors of time and orientation were considered orthogonal and fixed. The same

255

two-factor design was considered to test for any signiﬁcant differences in the mean

256

coverages via RM-permutational analysis of variance (RM-PERMANOVA). Univariate

257

analyses were performed using SPSS.13© according to the guidelines of Pardo and Ruiz

258

259

[89].

260

For the multivariate analyses, square root transformations were applied to the data,

261

and the analyses were performed using the Bray-Curtis similarity. The Bray-Curtis

262

similarity matrix was used to generate a non-metric multidimensional scaling (nMDS)

263

analysis, and the Kruskal stress coefficient was calculated to test the ordination [90].

264

The multivariate analyses were performed using the PRIMER6 software (complete with

265

266

267

the PERMANOVA+ package) [91-94].

# Results and discussion

268

The average covers for the five most abundant species (including the two pre-selected

269

sensitive target species *A. calycularis* and *P. clavata*) over the period of 2005 to 2014 at

270

271

the two locations (horizontal and vertical) are presented in Fig 3.

272

#### Fig 3. Graphic representation of the evolution of the cover of the main species

273

274

**observed at the monitoring stations.** H: horizontal; V: vertical.

275

Fig 4 illustrates the evolution of the covers of the sampled indicator species *A.*

276

*calycularis* and *P. clavata* at both locations (horizontal and vertical) together with the

277

time series of the background water temperatures in the sampling zones. These series

278

reflected no anomalous increases or decreases in temperature and only exhibited

279

oscillations that were attributable to seasonality. The covers of both species were clearly

280

greater in the vertical orientation than in the horizontal orientation. An increase and a

281

decrease were observed in the covers of *A. calycularis* and *P. clavata,* respectively, but

282

283

there were no significant differences.

284

#### Fig 4. Graphic representation of the evolution of the cover of the two main indicator

285

**species (*A. calycularis* and *P. clavata*), together with the bottom water temperature**

286

287

**time series.** The bars indicate the standard deviation. H: horizontal; V: vertical.

288

RM-ANOVA of the cover of each species in each of the two orientations (Table

289

1) indicated significant differences (p<0.05) in the covers of *Mesophyllum* sp. and

290

*Salmacina incrustans* in the shady (vertical) location over the time period. Regarding the

291

sunny (horizontal) location, the covers of *Mesophyllum* sp. and *Crambe* sp. exhibited

292

significant differences (p<0.05). No significant differences (p>0.05) were detected in the

293

294

covers of the remaining species.

295

#### Table 1. Results of one-way repeated measures ANOVA on coverage of each species

296

#### either in vertical and horizontal substrata.

297

***A. calycularis***

***P. clavata***

***Crambe* sp.	*Eunicella* sp.	*Mesophyllum* sp.	*P. fascialis***

***S. incrustans***

**Source of variation**

**Vertical Time^+^ Residuals**

**Mauchly's test of sphericity Transformation**

**Horizontal Time^+^ Residuals**

**Mauchly's test of sphericity Transformation**

**df**

**F**

**P***

**F**

**P***

**F**

**P***

**F**

**P***

**F**

**P***

**F**

**P***

**F**

**P***

9

10

1.159^2^

0.335

1.647^2^

0.171

0.793^2^

0.557

0.632^1^

0.359

3.474^2^

**0.018**

2.735^2^

0.058

2.931^2^	**0.021**

p<0.001

none

p<0.001

none

p<0.05

none

p=0.305

none

p<0.01

none

-

none

-

none

9

10

0.671^1^	0.277

0.593^1^	0.446

5.720^2^	**0.002**

1.749^2^	0.157

0.910^1^

**0.006**

1.087^2^	0.365

0.890^2^	0.426

p=0.490

none

p=0.052

none

p<0.01

none

p<0.001

none

p=0.101

none

-

none

-

none

298

* Greenhouse-Geisser correction was used when the spherity condition was not verified.

299

^+^Time correspond with years from 2005 to 2014.

300

1 Pillai´s trace.

301

302

303

304

305

2 Greenhouse-Geisser F

13

306

The RM-PERMANOVA analysis indicated significant differences in the

307

coralligenous assemblages (p<0.05) for the orientation factor (*i.e.*, vertical vs. horizontal)

308

but not for the time factor. The interaction of these two factors was also not significant

309

310

(Table 2).

311

Table 2**:** Results of repeated measures PERMANOVA.

| **Source** | **df** | **SS** | **MS** | **Pseudo-F** | **P(perm)** | **Unique perms** |
| --- | --- | --- | --- | --- | --- | --- |
| **Time** | 9 | 2150.5 | 238.95 | 2.4353 | 0.079 | 998 |
| **Orientation** | 1 | 30338 | 30338 | 309.2 | **0.001** | 998 |
| **Ti x Or** | 9 | 1207.9 | 134.21 | 1.3678 | 0.359 | 999 |
| **Res** | 60 | 5887.1 | 98.118 |  |  |  |
| **Total** | 79 | 39583 |  |  |  |  |

312

**Transformation**

None

313

314

The nMDS analysis (Fig 5) revealed a clear differentiation of the values between the

315

316

vertical and horizontal orientations.

317

#### Fig 5. Graphic representation of the non-metric MDS analysis for the whole

318

319

#### monitoring quadrats.

320

**Sampling procedure and analysis of the submarine images**

321

Previous studies have indicated that fixed monitoring stations constitute one of

322

the most robust methods for detecting changes in benthic communities over time [63, 95-

323

98]. An area of at least 1 m^2^ is sufficient for integrating various colonies and individuals

324

of diverse fauna in a single sample, and such diversity is a typical characteristic of benthic

325

rocky bottom communities [63, 99]. However, the definitive sampling grid size must be

326

defined by the final objective of the study [100]. In our case, the objective was not an

327

exhaustive description of the existing community but rather the evaluation of possible

328

changes in the covers of sessile target species within the community. Similarly, the

329

number of quadrats or replicates used must be defined by the target objectives. In other

330

studies of rocky bottoms or surfaces, three [34, 85, 101], five [102], six [103], and eight

331

replicates [104] have been employed. In all of these studies, the replicates had areas of

332

333

334

significantly less than 1 m^2^ with the exceptions of the studies by Fraschetti *et al.* [101] and Piazzi *et al.* [34] who also used areas of 1 m^2^.

335

Image analysis based on a monitoring system comprises a non-invasive method

336

that does not interfere with the natural development or evolution of the studied

337

community [63, 84, 96, 105, 106]. This method also allows for rapid data collection and

338

permanent data record generation. The images were captured using videos rather than

339

photographs mainly due to video’s greater speed and versatility when obtaining data [107-

340

109]. Video was thus time-saving and consequently allowed for the optimisation of diving

341

operations, which was very important given the depth at which the study was performed

342

343

and the number of replicates that were monitored.

344

## Choices of high biodiversity habitats, target species, and other

345

## companion organisms

346

In general, sublittoral marine habitats are characterised by diversity that increases

347

with depth [60, 110, 111]. Deep communities are dominated by animals, are better

348

structured, and exhibit less abrupt dynamics and smaller temporal changes than shallow

349

communities or those dominated by algae [60]. Additionally, as pointed out by

350

Ballesteros [112], within the scope of marine protected areas (MPAs), the selection of a

351

limited number of representative and/or key species from such communities is a sound

352

353

strategy for aiding their understanding and management.

354

Specific fixed benthic organisms have previously been used as indicators for the

355

monitoring of various environmental parameters; such species have been used as

356

indicators of global warming and climate change [57, 78, 113], sea level fluctuations [114,

357

358

115], and the influence of recreational diving on MPAs [56, 74, 116, 117].

359

Moreover, the use of representative taxa as proxies for entire communities has

360

proven to be a reliable alternative for evaluating the state of rocky bottom communities

361

comprising algae because the loss of information associated with the identification of

362

only some specific taxa does not greatly alter the results compared with results obtained

363

with complete datasets based on entire communities [3]. In hard bottom invertebrate

364

communities, it has also been found that sampling efforts can be reduced without

365

significant losses of information via the selection of indicator and representative species

366

367

[118].

368

*A. calycularis*, *P. clavata*, and *Eunicella* sp. (which were clearly more abundant

369

in the monitored vertical enclaves) play an important structural role in coralligenous

370

assemblages. These species colonise both horizontal bottoms and vertical walls and offer

371

ideal habitats for numerous other organisms [41, 119, 120]. Several previous studies of

372

coralligenous zones in different areas of the Mediterranean, such as the coasts of Italy and

373

France, have determined that the facies of *Paramuricea* and *Eunicella* are distinctive [76,

374

375

121].

376

None of these species exhibited significant cover alterations over the entire

377

monitoring period. Although *P. clavata* and *Eunicella sp*. have similar biological and

378

ecological characteristics, including similar rates of growth and production [122-124],

379

this similarity does not extend to their turnover rates. For *P. clavata,* this rate oscillates

380

between 7 and 9 years [122, 124], whereas for *Eunicella* sp., the rate is significantly lower

381

with a range of 3 to 4 years [123]. For this reason, during the long-term temporal

382

monitoring of fixed, limited points, we would expect *Eunicella* sp. to exhibit greater

383

oscillations in cover percentages than those that we observed due to mortality among the

384

colonies within the monitoring quadrats. Nevertheless, this phenomenon was not recorded

385

in our work, possibly because the magnitude of the studied area allowed for the

386

387

replacement of the dead organisms by new recruits to buffer the potential differences.

388

Also, it is important to point out that the two main indicator species, *A. calycularis*

389

and *P. clavata* (particularly the latter), are sensitive to changes in normal temperature

390

conditions [113, 125, 126, 127], although they both withstand seasonal fluctuations well

391

392

[112].

393

The differences found in the covers of certain species can also be explained by

394

natural processes. *Mesophyllum alternans* is among the organisms responsible for the

395

greatest percentage of cover (particularly in the horizontal orientation), but it is

396

considered to be opportunistic [45], which might explain any oscillations in cover in the

397

absence of any significant alterations to the reference species. The other two species that

398

exhibited significant differences, *i.e.*, *Salmacina incrustans* and *Crambe* sp., are

399

organisms that account for very low percentages of the overall cover. Variations in

400

abundances in deep rocky bottom communities are usually much more noticeable among

401

minor species than among the highly abundant or characteristic species [128]. In any case,

402

these two species can be classified as tolerant and relatively resistant to environmental

403

404

impacts [41].

405

Recent studies of coralligenous areas have only used vertical enclaves [121], but,

406

despite comprising the same species, the biological communities that occur at the two

407

orientations are clearly different due to the varying percentages of cover formed by those

408

species. Encrusting algae dominate horizontal enclaves, whereas populations of *P.*

409

*clavata* and *A. calycularis* are more abundant on vertical walls; these differences have

410

previously been mentioned by other authors [112, 129]. Therefore, wherever possible,

411

stations should be set up in both orientations, although vertical coralligenous enclaves

412

provide more information because they have greater biodiversity, are better structured

413

414

spatially and trophically, and have greater numbers of sensitive colonial species.

415

## Applications of fixed quadrats for the monitoring of rocky

416

## bottom habitats

417

The method tested here would be particularly useful for the WFD/MSFD in

418

relation to the environmental control of littoral zones based on biological indicators. The

419

WFD/MSFD has very few methodological tools for monitoring sessile benthic

420

invertebrates associated with hard substrates, although the tools that do exist are excellent

421

[130]. The environmental information provided by these species is significant because it

422

covers a wide range of sessile organisms that are long-lived and sensitive to changes in

423

the environment. These species can be used as reliable ecological sentries that keep watch

424

over the quality of littoral environments because they are not able to flee or be displaced

425

(as adults) when conditions deteriorate or significantly change.

426

427

The effectiveness of this monitoring method was demonstrated over the 10-year period

428

used to obtain a complete quantitative temporal series. As indicated by Gatti [76,131],

429

such series are very useful for evaluating both reference conditions and environmental

430

changes in ecosystems. Indeed, the main macrobiota species in the sublittoral community

431

432

in this study were stable and remained in excellent condition from 2005 to 2014.

433

The use of images is advantageous because, unlike the removal of physical

434

samples by scraping, it is neither destructive nor aggressive to the surroundings. The

435

scraping approach is not effective as a monitoring method in zones in which the fauna is

436

distributed in patches because it entails the destruction of relatively large areas [99]. In

437

protected locations and areas of special interest, this scraping option is particularly

438

439

undesirable.

440

The present image-based monitoring method is “low cost” in addition to being

441

“low effort” and requires minimal maintenance (repairs or replacements of the fixed

442

quadrats were necessary only twice over the 10-year monitoring period). Panayotidis [21]

443

indicated that the use of simple monitoring programs (*i.e.*, those involving taxonomic

444

efforts that are limited to representative species and simple statistical treatments) and low

445

446

budget methods is demonstrably effective for other rocky bottom communities.

447

According to recent studies, the minimum replication area required for

448

biodiversity studies of communities dominated by *Paramuricea clavata* [132], as well as

449

other hard-substrate benthic invertebrates [84, 133, 134], is less than the area used in the

450

replicates in our present work. Additionally, the captured images remain available for

451

subsequent, more detailed descriptions of the community. Higher quality images facilitate

452

the visual identification of more of the macrobenthic species that are present in the

453

community, and the system of photo-quadrats is more efficient than *in situ* visual census

454

455

methods [84] while simultaneously avoiding the destruction of the monitored areas.

456

As a final summary, Table 3 presents a comparison of the data from the monitoring

457

area of the present study with the data from several other studies of coralligenous bottoms

458

in terms of time and depth. Accounting for both area and time, the present study represents

459

460

a long-term monitoring of coralligenous assemblages.

461

#### Table 3: Comparison of the data of monitoring area, time and depth of several

462

#### studies on coralligenous bottoms.

|  | **Study**  [128] | **Area (m2)**  0.132 | **Depth (m)**  20 | **Time (years)**  2 |
| --- | --- | --- | --- | --- |
|  | [45] | 1.085 | 15-19 | 2 |
|  | [42] | 0.4 | 27 | 21 |
|  | [60] | 0.372 | 17-20 | 2 |
|  | [135] | 33.6 | 10-35 | 1 |
|  | [104] | 13.25 | 6-8 | 1 |
|  | [79] | 25 | 4-5 | 1 |
|  | [34] | 180 | 30-40 | 1 |
|  | [84] | 4.96 | 0-50 | 1 |
|  | Present study | 8 | 27-30 | 10 |
| 463 |  |  |  |  |

464

Finally, the proposed methodology could be extended to the entire geographical

465

area over which the target species of this study are representative of the rocky bottom

466

benthic communities that occur at similar depths and share similar biotic structures. This

467

methodology could also be applied in other areas in which target species can be

468

established and selected by employing the aforementioned implementation criteria.

469

Additionally, the SBPQ method involves minimal implementation difficulty both in terms

470

of cost and installation. The *a priori* selection of the target species simplifies the

471

taxonomic difficulty of visual identification. Therefore, this environmental warning and

472

surveillance system is not only oriented toward the scientific and technical communities

473

of the relevant coastal countries but also toward environmental volunteers associated with

474

diving centres and clubs. This methodology ultimately entails the aim of setting up

475

geographical networks in regions with sufficiently high levels of homogeneity (*e.g.*, the

476

Alboran Sea or, on a larger scale, the western Mediterranean). Citizen science initiatives

477

focusing on the mapping and monitoring of coralligenous assemblages have recently been

478

implemented [136]. In the future, these networks could provide early warnings of changes

479

to structured and unpolluted systems, particularly those in littoral zones close to places

480

subjected to strong anthropogenic pressure, which could be applied to the detection of

481

local or general environmental alterations (*i.e.*, climate change). In contrast, the nature of

482

the method, which is designed to monitor a group of taxa with respect to the total taxa of

483

the community, implies that it is not useful as such in the ecological study of these habitats

484

because the analysed information is biased. However, the generated databases (*i.e.*, stored

485

photographs) will allow for the performance of in-depth studies of the dynamics of the

486

species within the communities over time, assuming that significant changes can be

487

detected, and could aid parallel projects that aim to increase our knowledge of

488

489

490

coralligenous assemblages.

# Conclusions

491

The study validated the Sessile Bioindicators in Permanent Quadrats (SBPQ)

492

underwater environmental alert method in relation to the monitoring of pre-selected

493

sensitive and sessile benthic species (*P. clavata* and *A. calycularis*) associated with rocky

494

coralligenous habitats (which exhibit high stability and biodiversity) via the use of

495

permanent quadrats fixed on rocky shores in underwater SBPQ sentinel stations. The

496

obtained results confirmed the untested part of the method based on the initial hypothesis

497

that, over a long temporal duration (a ten-year period in this study) and in a highly

498

structured and biodiverse coralligenous assemblage, the cover of sensitive sessile species

499

does not change over time if the environmental stability characterising the habitat is not

500

altered. This stability of the cover of the sensitive sessile species is a key aspect for

501

confirming the reliability and robustness of the SBPQ method. Given that, as in the

502

present study, the selected species are very sensitive to increases in temperature and

503

deterioration of the environmental quality of the water column, the SBQP method is

504

useful as an underwater environmental alert system because it should be solely sensitive

505

to changes in the coverages of such species that result from physico-chemical changes in

506

the system. Such changes include gradual increases in temperature due to global warming

507

508

509

and changes due to the introduction of exotic species.

# Acknowledgments

510

This project was supported by the Regional Activity Centre for Special Protected

511

Areas (RAC/SPA) with the express aim of, among other things, achieving methodological

512

advances based on information provided by bioindicator species or communities

513

established on hard substrates within infralittoral zones. This paper also benefited from

514

infrastructures acquired within the framework of environmental research project number

515

1889, which was co-financed by the Port Authority of Seville, the Aquarium of Seville,

516

517

518

519

and the University of Seville.

520

521

# References

522

1. Gatti G, Bianchi C N, Morri C, Montefalcone M, Sartoretto S. Coralligenous reefs

523

state along anthropized coasts: Application and validation of the COARSE index,

524

based on a rapid visual assessment (RVA) approach. Ecol Indic. 2015; 52: 567-

525

526

576.

527

2. Gatti, G., Bianchi, C. N., Montefalcone, M., Venturini, S., Diviacco, G. Morri, C.

528

Observational information on a temperate reef community helps understanding

529

the marine climate and ecosystem shift of the 1980–90s. Mar Pollut Bull. 2017;

530

531

114: 528-538.

532

3. Puente A, Juanes J A. Testing taxonomic resolution, data transformation and

533

selection of species for monitoring macroalgae communities. Estuar Coast Shelf

534

535

Sci. 2008; 78: 327-340.

536

4. Warwick	R	M. Environmental	impact	studies	on	marine	communities:

537

538

pragmatical considerations. Australian Journal of Ecology. 1993; 18: 63-80.

539

5. Pearson T H, Rosenberg R. Macrobenthic succession in relation to organic

540

enrichment and pollution of the marine environment. Oceanography and Marine

541

542

Biology: An Annual Review. 1978; 16: 229–311.

543

6. Rygg B. Distribution of species along pollution-induced diversity gradients in

544

benthic communities in Norwegian fjords. Mar Pollut Bull. 1985; 16: 469–474.

| 545 |  | |
| --- | --- | --- |
| 546 | 7. | Ros J D, Cardell M J. Effect on benthic communities of a major input of organic |
| 547 |  | matter and other pollutants (coast off Barcelona, western Mediterranean). Environ |
| 548 |  | Toxicol Chem. 1991; 31–32: 441–450. |
| 549 |  |  |
| 550 | 8. | Dauer D M. Biological criteria, environmental health and estuarine macrobenthic |
| 551 |  | community structure. Mar Pollut Bull. 1993; 26: 249–257. |
| 552 |  |  |
| 553 | 9. | Estacio F, García-Adiego E, Fa D, García-Gómez J C, Daza J L, Hortas F, et al. |
| 554 |  | Ecological analysis in a polluted area of Algeciras Bay (Southern Spain): external |
| 555 |  | vs. internal outfalls and environmental implications. Mar Pollut Bull. 1997; 34- |
| 556 |  | 10: 780-793. |
| 557 |  |  |
| 558 | 10. | Estacio F, García-Adiego E M, Carballo J L, Sánchez-Moyano J E, García-Gómez |
| 559 |  | J C. Interpreting temporal disturbances in an estuarine benthic community under |
| 560 |  | combined anthropogenic and climatic effects. J Coast Res. 1999; 15-1: 155-167. |
| 561 |  |  |
| 562 | 11. | Sánchez-Moyano J E, García-Adiego E M, Estacio F. García-Gómez J C. Effects |
| 563 |  | of environmental factors on the spatial variation of the epifaunal polychaetes of |
| 564 |  | the algae *Halopteris scoparia* in Algeciras Bay (Strait of Gibraltar). |
| 565 |  | Hydrobiologia. 2002; 470: 133-148. |
| 566 |  |  |
| 567 | 12. | Guerra-García J M, García-Gómez J C. Crustacean assemblages and sediment |
| 568 |  | pollution in an exceptional case study: a harbour with two opposing entrances. |
| 569 |  | Crustaceana. 2004; 77-3: 353-370. |

570

571

13. Majeed S A. Organic matter and biotic indices on the beaches of North Britanny.

572

573

Mar Pollut Bull. 1987; 18: 490–495.

574

14. Grall J, Glémarec M. Using biotic indices to estimate macrobenthic community

575

576

perturbations in the Bay of Brest. Estuar Coast Shelf Sci. 1997; 44: 43–53.

577

15. Borja A, Franco J, Pérez V. A marine biotic index to establish the ecological

578

quality	of	soft-bottom	benthos	within	European	estuarine	and	coastal

579

580

environments. Mar Pollut Bull. 2000; 40: 1100–1114.

581

16. Simboura N, Zenetos A. Benthic indicators to use in ecological quality

582

classification of Mediterranean soft bottom marine ecosystems including a new

583

584

biotic index. Mediterr Mar Sci. 2002; 3: 77–111.

585

17. Rosenberg R, Blomqvist M, Nilsson H C, Cederwall H, Dimming A. Marine

586

quality assessment by use of benthic species abundance distributions: a proposed

587

new protocol within the European Union Water Framework Directive. Mar Pollut

588

589

Bull. 2004; 49: 728–739.

590

18. EC. Directive 2000/60/EC of the European Parliament and of the Council of 23

591

October 2000 establishing a framework for Community action in the field of water

592

593

policy. Official Journal of the European Union. 22/12/2000; L 327: 0001 – 0073.

594

19. EC. Directive 2008/56/EC of the European Parliament and of the Council of 17

595

June 2008 establishing a framework for community action in the field of marine

596

environmental policy (Marine Strategy Framework Directive). Official Journal of

597

598

the European Union. 25.6.2008; L 164: 19–40.

599

20. Orfanidis S, Panayotidis P, Stamatis N. Ecological evaluation of transitional and

600

coastal waters: a marine benthic macrophytes-based model. Mediterr Mar Sci.

601

602

2001; 2: 45–65.

603

21. Panayotidis P, Montesanto B, Orfanidis S. Use of low-budget monitoring of

604

macroalgae to implement the European Water Framework Directive. Journal of

605

606

Applied Phycology. 2004; 16: 49-59.

607

22. Ballesteros E, Torras X, Pinedo S, García M, Mangialajo L, Torres M. A new

608

methodology based on littoral community cartography dominated by macroalgae

609

for the implementation of the European Water Framework Directive. Mar Pollut

610

611

Bull. 2007; 55: 172–180.

612

23. Wells E, Wilkinson M, Wood P, Scanlan C. The use of macroalgal species

613

richness and composition on intertidal rocky seashores in the assessment of

614

ecological quality under the European Water Framework Directive. Mar Pollut

615

616

Bull. 2007; 55: 151–161.

617

24. Juanes J A, Guinda X, Puente A, Revilla J A. Macroalgae, a suitable indicator of

618

the ecological status of coastal rocky communities in the NE Atlantic. Ecol Indic.

619

620

2008; 8: 351-359.

621

25. Gaspar R, Pereira L, Neto J M. Ecological reference conditions and quality states

622

of marine macroalgae sensu Water Framework Directive: An example from the

623

intertidal rocky shores of the Portuguese coastal waters. Ecol Indic. 2012; 19: 24-

624

625

38.

626

26. Guinda X, Juanes J A, Puente A, Echavarri-Erasun B. Spatial distribution pattern

627

analysis of subtidal macroalgae assemblages	by a non-destructive rapid

628

629

assessment method. J Sea Res. 2012; 67: 34–43.

630

27. Neto J M, Gaspar R, Pereira L, Marques J C. Marine Macroalgae Assessment

631

Tool (MarMAT) for intertidal rocky shores. Quality assessment under the scope

632

633

of the European Water Framework Directive. Ecol Indic. 2012; 19: 39-47.

634

28. Krause-Jensen D, Greve T M, Nielsen K. Eelgrass as a bioindicator under the

635

European Water Framework Directive*.* Water Resources Management. 2005; 19:

636

637

63–75.

638

29. Foden J. Assessment metrics for littoral seagrass under the European Water

639

Framework Directive; outcomes of UK intercalibration with the Netherlands.

640

641

Hydrobiologia. 2007; 579: 187–197.

642

30. Foden J, Brazier D P. Angiosperms (seagrass) within the EU Water Framework

643

644

Directive: A UK perspective. Mar Pollut Bull. 2007; 55: 181–195.

645

31. Deter J, Descamp P, Ballesta L, Boissery P, Holon F. A preliminary study toward

646

an index based on coralligenous assemblages for the ecological status assessment

647

648

of Mediterranean French coastal waters. Ecol Indic. 2012; 20: 345-352.

649

32. Canovas-Molina A, Montefalcone M, Bavestrello G, Cau A, Bianchi C N, Morri

650

C, et al. A new ecological index for the status of mesophotic megabenthic

651

assemblages in the Mediterranean based on ROV photography and video footage.

652

653

Cont Shelf Res. 2016; 121: 13–20.

654

33. Ferrigno F, Russo G F, Sandulli R. Coralligenous Bioconstructions Quality Index

655

(CBQI): a synthetic indicator to assess the status of diﬀ erent types of

656

657

coralligenous hábitats. Ecol Indic. 2017; 82: 271–279.

658

34. Piazzi L, Gennaro P, Cecchi E, Serena F, Bianchi C N, Morri C, et al. Integration

659

of ESCA index through the use of sessile invertebrates. Sci Mar. 2017; 81-2: 283-

660

661

290.

662

35. Sartoretto S, Schohn T, Nike Bianchi C, Morri C, Garrabou J, Ballesteros E, et al.

663

An integrated method to evaluate and monitor the conservation state of

664

coralligenous habitats: The INDEX-COR approach. Mar Pollut Bull. 2017; 120:

665

666

222–231.

667

36. Enrichetti F, Bo M, Morri C, Montefalcone M, Toma M, Bavestrello G, et al.

668

Assessing the environmental status of temperate mesophotic reefs: A new,

669

670

integrated methodological approach. Ecol Indic. 2019; 102: 218–229.

671

37. Piazzi L, Cecchi E, Gennaro P, Morri C, Montefalcone M, Serena F, et al. STAR:

672

An integrated and standardized procedure to evaluate the ecological status of

673

674

coralligenous reefs. Aquat Conserv. 2019; 29: 189–201.

675

38. Carballo J L, Naranjo S A, García-Gómez J *C.* Use of marine sponges as stress

676

indicators in marine ecosystems at Algeciras Bay (southern Iberian Peninsula).

677

678

Mar Ecol Prog Ser. 1996; 135 :109-122.

679

39. Naranjo S A, Carballo J L, Garcia-Gómez J C. Effects of environmental stress on

680

ascidian populations in Algeciras Bay (southern Spain). Possible marine

681

682

bioindicators? Mar Ecol Prog Ser. 1996; 144: 119-131.

683

40. García-Gómez J C. Biota Litoral y Vigilancia Ambiental en las Áreas Marinas

684

685

Protegidas. Junta de Andalucía, Consejería de Medio Ambiente. 2007.

686

41. García-Gómez J C. A guide on environmental monitoring of rocky seabeds in

687

Mediterranean Marine Protected Areas and surrounding zones. Marine Biology

688

Laboratory, Department of Zoology, Faculty of Biology, University of Seville.

689

R+D+I Biological Research Area, Seville Aquarium. Ed. RAC/ SPA -

690

MedMPAnet Project, Tunis. 2015.

691

http://www.rac-

692

spa.org/sites/default/files/doc_medmpanet/final_docs_regional/78.guide_suivi_a

693

mp_med_en.pdf

694

695

(Available for free download).

696

42. Garrabou J, Harmelin, J G. A 20-year study on life-history traits of a harvested

697

long-lived temperate coral in the NW Mediterranean: insights into conservation

698

699

and management needs. Journal of Animal Ecology. 2002; 71: 966–978.

700

43. Coma R, Serrano E, Linares C, Zabala M, Ribes M. Effect of a severe storm event

701

on the mortality rate of the gorgonian *Paramuricea clavata* on the Medes Islands

702

Marine Reserve and the nearby Montgrí coast. In: Mateo, M. A. and García-

703

Rubies, T. (Eds.), Assessment of the ecological impact of the extreme storm of

704

Sant Esteve´s Day (26 December 2008) on the littoral ecosystems of the north

705

Mediterranean Spanish coasts. Final Report (PIEC 200430E599). Centro de

706

Estudios Avanzados de Blanes, Consejo Superior de Investigaciones Científicas,

707

708

Blanes, Spain, 2012; 67-78.

709

44. Garrabou J. Life-history traits of *Alcyonium acaule* and *Parazoanthus axinellae*

710

(Cnidaria, Anthozoa), with emphasis on growth. Mar Ecol Prog Ser. 1999; 178:

711

712

193-204.

713

45. Garrabou J, Ballesteros E. Growth of *Mesophyllum alternans* and *Lithophyllum*

714

*frondosum* (Corallinales, Rhodophyta) in the northwestern Mediterranean*.* Eur J

715

Phycol. 2000; 35: 1–10.

716

717

46. Glynn P W. Coral Reef Bleaching in the 1980s and Possible Connections with

718

719

Global Warming. TREE. 1991; 6-6: 175-178.

720

47. Walther G R, Post E, Convey P, Menzel A, Parmesan C, Trevor J C, et al.

721

722

Ecological responses to recent climate change. Nature. 2002; 412: 389-395.

723

48. Baker A C, Glynn P W, Riegl B. Climate change and coral reef bleaching: An

724

ecological assessment of long-term impacts, recovery trends and future outlook.

725

726

Estuar Coast Shelf Sci. 2008; 80: 435–471.

727

49. Jordà G, Marbà N, Duarte C M. Mediterranean seagrass vulnerable to regional

728

729

climate warming. Nat Clim Chang. 2012; 2: 821-824.

730

50. Dukes J S, Mooney H. Does global change increase the success of biological

731

732

invaders? Trends Ecol Evol. 1999; 14: 135–139.

733

51. Occhipinti-Ambrogi A. Global change and marine communities: Alien species

734

735

and climate change. Mar Pollut Bull. 2007; 55: 342–352.

736

52. Soto C. The potential impacts of global climate change on marine protected areas.

737

738

Rev Fish Biol Fish. 2002; 11: 181–195.

739

53. Carvalho S B, Brito J C, Crespo E G, Watts M E, Possingham H P. Conservation

740

planning under climate change: Toward accounting for uncertainty in predicted

741

species distributions to increase confidence in conservation investments in space

742

743

and time. Biol Conserv. 2011; 144: 2020–2030.

744

54. Hawkins S J. Marine conservation in a rapidly changing world. Aquat Conserv.

745

746

2012; 22: 281–287.

747

55. Romano J C, Bensoussan N, Younes W A N, Arlhac D. Anomalie thermique dans

748

les eaux du golfe de Marseille durant l’été 1999. Une explication partielle de la

749

mortalité d’invertébrés fixés? Comptes Rendus de l’Académie des Sciences de

750

751

Paris, Sciences de la vie / Life Sciences. 2000; 323: 415–427.

752

56. Linares C, Coma R, Diaz D, Zabala M, Hereu B, Dantart L. Immediate and

753

delayed effects of a mass mortality event on gorgonian population dynamics and

754

benthic community structure in the NW Mediterranean Sea. Mar Ecol Prog Ser.

755

756

2005; 305:127–137.

757

57. Bensoussan N, Romano J C, Harmelin J G, Garrabou J. High resolution

758

characterization of northwest Mediterranean coastal waters thermal regimes: To

759

better understand responses of benthic communities to climate change. Estuar

760

761

Coast Shelf Sci. 2010; 87: 431–441.

762

58. Foster M S, Harrold C, Hardin D D. Point vs. photo quadrat estimates of the cover

763

764

of sessile marine organisms. J Exp Mar Bio Ecol. 1991; 146: 193-203.

765

59. Roberts D E, Fitzhenry S R, Kennelly S J. Quantifying subtidal macrobenthic

766

assemblages on hard substrata using a jump camera method. J Exp Mar Bio Ecol.

767

768

1994; 177: 157-170.

769

60. Garrabou J, Ballestero E, Zabala M. Structure and Dynamics of North-western

770

Mediterranean Rocky Benthic Communities along a Depth Gradient. Estuar Coast

771

772

Shelf Sci. 2002; 55: 493–508.

773

61. Leujak W, Ordmon R F G. Comparative accuracy and efficiency of six coral

774

775

community survey methods. J Exp Mar Bio Ecol. 2007; 351-1-2: 168-187.

776

62. Van Rein H, Brown C J, Schoeman D S, Quinn R, Breen J. Fixed-station

777

monitoring of a harbour wall community: the utility of low-cost photomosaics and

778

779

scuba on hard-substrata. Aquat Conserv. 2011; 21: 690–703.

780

63. Van Rein H, Schoeman D S, Brown C J, Quinn R, Breen J. Development of

781

benthic monitoring methods using photoquadrats and scuba on heterogeneous

782

hard-substrata: a boulder-slope community case study. Aquat Conserv. 2011; 21-

783

784

7: 676–689.

785

64. Van Rein H, Schoeman D S, Brown C J, Quinn R, Breen J. Development of low-

786

cost image mosaics of hard-bottom sessile communities using SCUBA:

787

comparisons of optical media and of proxy measures of community structure. J

788

789

Mar Biol Assoc U.K. 2012; 92-1: 49-62.

790

65. Parravicini V, Micheli F, Montefalcone M, Morri C, Villa E, Castellano M, et al.

791

Conserving Biodiversity in a Human-Dominated World: Degradation of Marine

792

Sessile Communities within a Protected Area with Conflicting Human Uses.

793

794

PLoS One. 2013; 8-10: e75767.

795

66. Alemany J. Puerto Bahía Algeciras. Entre dos mares y dos continentes. 100 años

796

797

de historia. Lunwerg Eds. 2005.

798

67. Morales C. Caracterización de la calidad de sedimentos afectados por vertidos de

799

petróleo: comparación entre casos de vertidos accidentales (impacto agudo) frente

800

a derrames continuos (impacto crónico). Tesis Doctoral. Universidad de Cádiz,

801

802

Facultad de Ciencias del Mar y Ambientales. 2007.

803

68. Consejería de Medio Ambiente de la Junta de Andalucía*.* Acuerdo específico entre

804

la Consejería de Medio Ambiente y la Universidad de Sevilla para el seguimiento

805

y la vigilancia ambiental de las comunidades rocosas intermareales y submareales

806

807

del parque natural del Estrecho. Tercer informe parcial. 2009.

808

69. Consejería de Medio Ambiente y ordenación del territorio. Red de información

809

ambiental de Andalucía.

810

[(http://www.juntade](http://www.juntadeandalucia.es/medioambiente/portal_web/rediam/documentos)a[ndalucia.es/medioambiente/portal_web/rediam/documentos](http://www.juntadeandalucia.es/medioambiente/portal_web/rediam/documentos)

811

812

/docs/litoral/Informe_resultados_sst.pdf).

813

70. Zibrowius H. The "southern" *Astroides calycularis* in the Pleistocene of the

814

northern Mediterranean - an indicator of climatic changes (Cnidaria, Scleractinia).

815

816

Geobios. 1995; 28: 9−1.

817

71. Benedetti-Cecchi L, Airoldi L, Abbiati M, Cinelli F. Exploring the causes of

818

spatial variation in an assemblage of benthic invertebrates from a submarine cave

819

820

with sulphur springs. J Exp Mar Bio Ecol. 1996; 208: 153-168.

821

72. Linares C, Coma R, Garrabou J, Díaz D, Zabala M. Size distribution, density and

822

disturbance	in	two	Mediterranean	gorgonians: *Paramuricea	clavata*	and

823

824

*Eunicella singularis*. Journal of Applied Ecology. 2008; 45: 688–699.

825

73. Cupido R, Cocito S, Barsanti M, Sgorbini S, Peirano A, Santangelo G.

826

Unexpected long-term population dynamics in a canopy-forming gorgonian coral

827

828

following mass mortality. Mar Ecol Prog Ser. 2009; 394: 95–200.

829

74. Linares C, Doak D F. Forecasting the combined effects of disparate disturbances

830

on the persistence of long-lived gorgonians: a case study of *Paramuricea clavata*.

831

832

Mar Ecol Prog Ser, 2010; 402: 59–68.

833

75. Terrón-Sigler A, Peñalver-Duque P, León-Muez D, Espinosa-Torre F. Spatio-

834

temporal macrofaunal assemblages associated with the endangered orange coral

835

*Astroides calycularis* (Scleractinia: Dendrophylliidae). Aquatic biology. 2014;

836

837

21: 143-154.

838

76. Gatti G, Bianchi C N, Morri C, Montefalcone M, Sartoretto S. Coralligenous reefs

839

state along anthropized coasts: Application and validation of the COARSE index,

840

based on a rapid visual assessment (RVA) approach. Ecol Indic. 2015; 52: 567-

841

842

576.

843

77. Casas-Güell E, Teixidó N, Garrabou J, Cebrian E. Structure and biodiversity of

844

coralligenous assemblages over broad spatial and temporal scales. Mar Biol.

845

846

2015; 162: 901–912.

847

78. Coma R, Linares C, Ribes M, Diaz D, Garrabou J, Ballesteros E. Consequences

848

of a mass mortality in populations of Eunicella singularis (Cnidaria: Octocorallia)

849

in Menorca (NW Mediterranean). Marine Ecology Progress Series. 2006; 327:

850

851

51–60.

852

79. Parravicini V, Morri C, Ciribilli G, Montefalcone M, Albertelli G, Bianchi, C N.

853

Size matters more than method: Visual quadrats vs photography in measuring

854

human impact on Mediterranean rocky reef communities. Estuar Coast Shelf Sci.

855

856

2009; 81: 359–367.

857

80. Tkachenko K S. An Evaluation of the analysis system of video transects used to

858

859

sample subtidal epibiota. J Exp Mar Bio Ecol. 2005; 318: 1-9.

860

81. Dethier M N, Graham E S, Cohen S, Tear L M. Visual versus random-point

861

percent cover estimations: 'objective' is not always better. Mar Ecol Prog Ser.

862

1993; 96: 93-100.

863

864

82. Dietz H, Steinlein T. Determination of plant species cover by means of image

865

866

analysis. J Veg Sci. 1996; 7: 131-136.

867

83. Leonard G H, Clark R P. Point quadrat versus video transect estimates of the cover

868

869

of benthic red algae. Mar Ecol Prog Ser. 1993; 101: 203-208.

870

84. Sant N, Chappuis E, Rodríguez-Prieto C, Real M, Ballesteros E. Cost-benefit of

871

three different methods for studying Mediterranean rocky benthic assemblages.

872

873

Sci. Mar. 2017; 81-1: 129-138.

874

85. Benedetti-Cecchi L, Airoldi L, Abbiati M, Cinelli F. Estimating the abundance of

875

benthic invertebrates: a comparison of procedures and variability between

876

877

observers. Mar Ecol Prog Ser. 1996; 138: 93-101.

878

86. J W. Significance test for sphericity of a normal n-variate distribution. Annals of

879

880

Mathematical Statistics. 1940; 11; 204-209.

881

87. Geisser S, Greenhouse S. An extension of Box´s results on the use of the F

882

distribution in multivariate analysis. Annals of Mathematical Statistics. 1958; 29:

883

884

885-891.

885

88. Greenhouse S, Geisser S. On methods in the analysis of profile data.

886

887

Psychometrika. 1959; 24: 95-112.

888

89. Pardo A, Ruiz M A. Análisis de varianza (III). El procedimiento Modelo Linear

889

General: Medidas repetidas. In: Sánchez-González, C. (Ed.). Análisis de datos con

890

SPSS 13 Base. McGraw-Hill / Interamericana de España S. A. U., Madrid. 2005;

891

892

395-427.

893

90. Kruskal J B, Wish M. Multidimensional Scaling. Sage University Paper Series on

894

Quantitative Applications in the Social Sciences, Sage Publications, Newbury

895

896

Park. 1978; No. 07-011.

897

91. Clarke K R, Gorley R N. PRIMER v6: User manual/tutorial. PRIMER-E Ltd.

898

899

Plymouth, UK. 2006.

900

92. Clarke K R, Somerfield P J, Airoldi L, Warwick R M. Exploring interactions by

901

902

second-stage community analyses. J Exp Mar Bio Ecol. 2006; 338: 179-192.

903

93. Anderson M J, Gorley R N, Clarke K R. PERMANOVA + for PRIMER. Guide

904

905

to software and statistical methods. Plymouth, UK: PRIMER-E Ltd. 2008.

906

94. Anderson, M. J. and Walsh, D. C. I. PERMANOVA, ANOSIM and the Mantel

907

test in the face of heterogeneous dispersions: What null hypothesis are you

908

909

testing? Ecol Monogr. 2013; 83: 557-574.

910

95. Lundalv T, Larsson C S, Axelsson L. Long-term trends in algal-dominated rocky

911

subtidal communities on the Swedish west coast - a transitional system?

912

Hydrobiologica. 1986; 142: 81–95.

913

914

96. Lanyon J M, Marsh H. Temporal changes in the abundance of some tropical

915

916

intertidal seagrasses in North Queensland. Aquat Bot. 1995; 49: 217-237.

917

97. Hill J, Wilkinson C. Methods for Ecological Monitoring of Coral Reefs.

918

919

Australian Institute of Marine Science: Townsville. 2004.

920

98. Molloy P P, Evanson M, Nellas A C, Rist J L, Marcus J E, Koldewey H J et al*.*

921

How much sampling does it take to detect trends in coral-reef habitat using

922

923

photoquadrat surveys? Aquat Conserv. 2013; 23: 820–837.

924

99. Cattaneo-Vietti R, Albertelli G, Bavestrello G, Bianchi C N, Cerrano C,

925

Chiantore, et al. Can rock composition affect sublittoral epibenthic communities?

926

927

PSZN: Marine Ecology. 2002; 23: 65–77.

928

100. Bianchi C N, Pronzato R, Cattaneo-Vietti R, Benedetti-Cecchi L, Morri C,

929

Pansini M, et al. Mediterranean marine benthos: a manual of methods for its

930

931

sampling and study. Hard bottoms. Biol Mar Mediterr. 2004; 11: 85–215.

932

101. Fraschetti S, Bianchi C N, Terlizzi A, Fanelli G, Morri C, Boero F. Spatial

933

variability and human disturbance in shallow subtidal hard substrate assemblages:

934

935

a regional approach. Mar Ecol Prog Ser. 2001; 212: 1–12.

936

102. Bevilacqua S, Terlizzi A, Fraschetti S, Russo G F, Boero F. Mitigating

937

human disturbance: can protection influence trajectories of recovery in benthic

938

939

assemblages? Journal of Animal Ecology. 2006; 75: 908–920.

940

103. Guidetti P, Bianchi C N, Chiantore M, Schiaparelli S, Morri C, Cattaneo-

941

Vietti R. Living on the rocks: substrate mineralogy and the structure of subtidal

942

rocky substrate communities in the Mediterranean Sea. Mar Ecol Prog Ser. 2004;

943

944

274: 57–68.

945

104. Bussotti S, Terlizzi A, Fraschetti S, Belmonte G, Boero F. Spatial and

946

temporal variability of sessile benthos in shallow Mediterranean marine caves.

947

948

Mar Ecol Prog Ser. 2006; 325: 109–119.

949

105. Bohnsack J A*.* Photographic quantitative sampling of hard-bottom benthic

950

951

communities*.* Bulletin of Marine Science. 1979; 29: 242-252.

952

106. Martí R, Uriz M J, Ballesteros E, Turon X. Seasonal variation in the

953

structure of three Mediterranean algal communities in various light conditions.

954

955

Estuar Coast Shelf Sci. 2005; 64: 613–622.

956

107. Whorff J S, Grifting L. A video recording and analysis system used to

957

958

sample intertidal communities. J Exp Mar Bio Ecol. 1992; 160: 1-12.

959

108. Magorrian B H, Service M. Analysis of Underwater Visual Data to

960

Identify the Impact of Physical Disturbance on Horse Mussel (*Modiolus*

961

962

*modiolus*) Beds. Mar Pollut Bull. 1998; 36: 354-359.

963

109. Cabaitan P C, Licuanan W Y, Gomez E D. Comparison between

964

videographic	and	photographic	methods	in	assessing	coral	reef	benthic

965

966

communities. Sci Diliman. 2007; 19: 7–13.

967

110. Jackson J B C. Adaptation and diversity of reef corals. Bioscience. 1991;

968

969

41: 472–482.

970

111. Witman J D, Grange, K R*.* Links between rain, salinity, and predation in a

971

972

rocky subtidal community. Ecology. 1998; 79: 2429–2447.

973

112. Ballesteros E*.* Mediterranean coralligenous assemblages: a synthesis of

974

present knowledge. Oceanography and Marine Biology: An Annual Review. 2006

975

976

44: 123-195.

977

113. Garrabou J, Coma R, Bensoussan N, Bally M, Chevaldonné P, Cigliano

978

M, et al. Mass mortality in Northwestern Mediterranean rocky benthic

979

communities: effects of the 2003 heat wave. Glob Chang Biol. 2009; 15: 1090–

980

981

1103.

982

114. Laborel	J,	Laborel-Deguen	F.	Sea-level	indicators,	biologic.	In:

983

Encyclopedia of Coastal Science (Ed. Schwartz, M.), Wiley, New York. 2005;

984

985

833-834.

986

115. Rovere A, Antonioli F, Bianchi C N. Fixed biological indicators. In:

987

Handbook of Sea-Level Research (Eds. Shennan, I., Long, A. J. and Horton, B.

988

989

P.). John Wiley & Sons, Ltd, Chichester, UK. 2015; 268-280.

990

116. Di Franco A, Milazzo M, Baiata P, Tomasello A, Chemello R. Scuba diver

991

behaviour and its effects on the biota of a Mediterranean marine protected area.

992

993

Environ Conserv. 2009; 36: 32–40.

994

117. Terrón-Sigler A, León-Muez D, Peñalver-Duque P, Espinosa-Torre F. The

995

effects of SCUBA diving on the endemic Mediterranean coral *Astroides*

996

997

*calycularis*. Ocean Coast Manag. 2016; 122: 1-8.

998

118. Urkiaga-Alberdi J, Pagola-Carte S, Saiz-Salinas J I*.* Reducing effort in the

999

1000

use of benthic bioindicators. Acta Oecologica. 1999; 20: 489-497.

1001

119. Sebens K P. Habitat structure and community dynamics in marine benthic

1002

systems. In: Bell, S. S., McCoy, E. D., Mushinsky H. R. (Eds) Habitat structure,

1003

1004

Chapman & Hall, London. 1991; 211–234.

1005

120. Orejas C, López-Gónzalez P J. Gorgonias y formaciones coralinas.

1006

Arquitectos de nuestros mares. In: Martínez, R. and Cornejo, J. M. (Eds.). Mares

1007

de España, Secretaría General del Mar, Dirección General de Sostenibilidad de la

1008

Costa y el Mar, Ministerio de Medio Ambiente y Medio Rural y Marino, Madrid.

1009

1010

2008.

1011

121. Piazzi L, Balata D, Cecchi E, Gennaro P, Serena F. Effectiveness of

1012

different investigation procedures in detecting anthropogenic impacts on

1013

1014

coralligenous assemblages. Sci. Mar. 2014; 78-3: 319-328.

1015

122. Mistri M, Ceccherelli V U. Growth and secondary production of the

1016

Mediterranean gorgonian *Paramuricea clavata*. Mar Ecol Prog Ser. 1994; 103:

1017

1018

291-296.

1019

123. Weinbauer M G, Velimirov B. Biomass and secondary production of the

1020

temperate gorgonian coral *Eunicella cavolini* (Coelenterata: Octocorallia). Mar

1021

1022

Ecol Prog Ser. 1995; 121: 211–216.

1023

124. Coma R, Ribes M, Zabala M, Gili J. M. Growth in a Modular Colonial

1024

1025

Marine Invertebrate. Estuar Coast Shelf Sci. 1998; 47: 459–470.

1026

125. Perez T, Garrabou J, Sartoretto S, Harmelin J G, Francour P, Vacelet J.

1027

Mortalité massive d’invertébrés marins: un événement sans précédent en

1028

Méditerranée nord-occidentale. Comptes Rendus de l´Académie des Sciences de

1029

1030

Paris, Sciences de la Vie. 2000; 323: 853–865.

1031

126. Bianchi	C	N.	Biodiversity	issues	for	the	forthcoming	tropical

1032

1033

Mediterranean Sea. Hydrobiologia. 2007; 580: 7–21.

1034

127. Lejeusne C, Chevaldonne P, Pergent-Martini C, Boudouresque C F, Pérez

1035

T. Climate change effects on a miniature ocean: the highly diverse, highly

1036

1037

impacted Mediterranean Sea. Trends Ecol Evol. 2010; 25-4: 250–260.

1038

128. Peckol P, Searles R B. Temporal and Spatial Patterns of Growth and

1039

Survival of Invertebrate and Algal Populations of a North Carolina Continental

1040

1041

Shelf Community. Estuar Coast Shelf Sci. 1984; 18: 133-143.

1042

129. True M. A. Étude quantitative de quatre peuplements sciaphiles sur

1043

substrat rocheux dans la région marseillaise. Bulletin Institute Océanographique

1044

1045

de Monaco. 1970; 1410: 1–48.

1046

130. Montefalcone M, Morri C, Bianchi C N, Bavestrello G, Piazzi L. The two

1047

facets of species sensitivity: Stress and disturbance on coralligenous assemblages

1048

1049

in space and time. Mar Pollut Bull. 2017; 117: 229-238.

1050

131. Gatti G, Bianchi C N, Montefalcone M, Venturini S, Diviacco G, Morri C.

1051

Observational information on a temperate reef community helps understanding

1052

the marine climate and ecosystem shift of the 1980–90s. Mar Pollut Bull. 2017;

1053

1054

114: 528-538.

1055

132. Kipson S, Fourt M, Teixidó N, Cebrian E, Casas E, Ballesteros E, et al.

1056

Rapid Biodiversity Assessment and Monitoring Method for Highly Diverse

1057

Benthic Communities: A Case Study of Mediterranean Coralligenous Outcrops.

1058

1059

PLoS One. 2011; 6-11: e27103.

1060

133. Weinberg S. The minimal area problem in invertebrate communities of

1061

1062

Mediterranean rocky substrata. Mar Biol. 1978; 49: 33–40.

1063

134. Gili	J	M, Ballesteros	E. Structure	of	cnidarian	populations	in

1064

Mediterranean sublittoral benthic communities as a result of adaptation to

1065

1066

different environmental conditions. Oecologia Aquatica. 1991; 10: 243–254.

1067

135. De Biasi A M, Bianchi C N, Aliani S, Cocito S, Peirano A, Dando P, et al.

1068

Epibenthic communities in a marine shallow area with hydrothermal vents.

1069

1070

Chemistry and Ecology. 2004; 20: 89–105.

1071

136. Gerovasileiou V, Dailianis T, Panteri E, Michalakis M, Gatti G, Sini M, et

1072

al. CIGESMED for divers: Establishing a citizen science initiative for the

1073

mapping and monitoring of coralligenous assemblages in the Mediterranean Sea.

1074

1075

Biodivers Data J. 2016; 4: e8692.

Figure	Click here to download Figure Fig 1.tif

Figure	Click here to download Figure Fig 2.tif

Figure	Click here to download Figure Fig 3.tif

Figure	Click here to download Figure Fig 4.tif

Figure	Click here to download Figure Fig 5.tif
