# Supplementary material for: Detect coastal disturbances and climate change effects in coralligenous community through sentinel stations
Source: PLoS One. 2020 May 5;15(5):e0231641. doi: 10.1371/journal.pone.0231641 (PMC7200002; doi:10.1371/journal.pone.0231641)
Supplement: Attachment — Submitted filename: Responses to reviewers.docx [file pone.0231641.s003.docx]

Responses to reviewers

Reviewer #1: I have incorporated your suggestions into my revision. Thank for your help. Below I present your numbered comments and corresponding response.

Comment 1:

The RAC-SPa manual cited (41) is a great work but the method it should be summarized and described for non-mediterranean scientist. I think it should be also of great importance to briefly introduce also the RAC-SPA and its work for non-Mediterranean scientist.

Answer to comment number 1:

The methodology has been expanded and a more detailed description of the SBPQ method has been included. In addition, two figures have been added to illustrate it. The RACSPA has also been briefly presented.

Comment 2:

The photographic method has been applied since at least 30 years from the use of the first
underwater photographic apparatus and the ''sensitive' species has been described since 1958 by Peres and Picard in their ' Manuel de bionomie bentonique' etc. etc..
I think that the introduction should be rewritten considering also that a lot of scientitst in these last years, have worked on the coralligenous and to the creation of indices. You cited many papers on the subject this but do not describe the differences between their approach and your.

I found also these papers:
-Julie Deter a,b,⁎, Pierre Descamp c, Pierre Boissery d, Laurent Ballesta c, Florian Holon cA rapid photographic method detects depth gradient in coralligenous assemblages.
Journal of Experimental Marine Biology and Ecology 418–419 (2012) 75–82
-Silvija Kipson1,3*, Maı¨a Fourt2, Nu´ ria Teixido´ 1,6, Emma Cebrian4, Edgar Casas1, Enric Ballesteros5, Mikel
Zabala6, Joaquim Garrabou. Rapid Biodiversity Assessment and Monitoring Method for Highly Diverse Benthic Communities: A Case Study of Mediterranean Coralligenous Outcrops PLoS ONE | www.plosone.org 1 November 2011 | Volume 6 | Issue 11 | e27103
-Giulia Gattia*, Monica Montefalconea, Alessio Rovereb, Valeriano Parravicinibc, Carla Morria, Giancarlo Albertellia and Carlo Nike Bianchi.Seafloor integrity down the harbor waterfront: the coralligenous shoals off Vado Ligure (NW Mediterranean)Advances in Oceanography and Limnology Vol. 3, No. 1, June 2012, 51–67
-Luigi Piazzi1 | Carlo Nike Bianchi2 | Enrico Cecchi3 | Giulia Gatti4 | Ivan Guala5 |
Carla Morri2 | Stéphane Sartoretto6 | Fabrizio Serena3,7 | Monica Montefalcone2 What's in an index? Comparing the ecological information provided by two indices to assess the status of coralligenous reefs in the NW Mediterranean SeaAquatic Conserv: Mar Freshw Ecosyst. 2017;27:1091–1100

Answer to comment number 2:

A paragraph has been included describing the differences between the proposed methodology and that designed by other authors. The articles suggested by the reviewer have been taken into account and cited.

Reviewer #2: I have incorporated your suggestions into my revision. Thank you for your contribution. It has been very useful. Below I present your numbered comments and corresponding response.

Comment 1:

I understand that the SBPQ method here proposed is simple, low-cost and easy to use, and I support the idea of a network of sentry stations monitored with fixed quadrats. Anyway, as already mentioned by the authors (line 420), other studies have recently provided excellent tools to monitor benthic ecosystems. Thus, a more robust justification to develop a new one should be included.

Answer to comment number 1:

As previously mentioned, a justification of the proposal has been included in the introduction section.

Comment 2:

Authors state that “the SBPQ method was proposed by García-Gómez [41], but its validation is pending a study of a long time series that could confirm that the coverages of long-cycle indicator species do not change over time in undisturbed benthic environments with high levels of biodiversity unless a significant environmental alteration is introduced into and modifies the system” (lines 162-166). I am not convinced that it is enough for the complete validation of the method. This study could prove that the basic idea of monitoring the cover of sensitive indicator species through time is useful, but not that the method itself is able to properly detect environmental changes.

Answer to comment number 2:

The citation of a recently published article has been included. This study shows the validity of the method to detect disturbances for invasive species.

García-Gómez J, Sempere-Valverde J, González A, Martínez-Chacón M, Olaya-Ponzone L, Sánchez-Moyano E, Ostalé Valriberas E, Megina C. From exotic to invasive in record time: the extreme impact of *Rugulopteryx okamurae* (dictyotales, Ochrophyta) in the strait of Gibraltar. Science of The Total Environment. 704 (2020) 135408.

Comment 3:

No other aspect of this method has been previously validated somewhere else through a peer review process. I suggest to include a more detailed description of the SBPQ methodology within the M&M section, providing not only information on target species and sampling procedure but also on the outputs processing, which are currently missing. I can see that in García-Gómez [41] a scale has been set out to detect significative environmental changes (<25%, no impact; 25-50%, orange warning sign; >50%, red warning sign; page 115). This is an interesting point and it has to be explained how these values have been selected. In my opinion, a loss of 25% cover in a species that cover on average 30-35% of the quadrant (like P. clavata does in the present study, Fig. 4) is more than considerable.

Answer to comment number 3:

The method has been described in detail and the references used to establish the defined coverage percentage classes have been included. In addition, the methodology was partially validated in the previously mentioned study (García-Gómez et al., 2020).

Comment 4:

The best way to validate this method is to compare different cases characterized by different ecological settings. This would be especially useful to set the scale proposed in García-Gómez [41]. Anyway, I understand that it is really complicated to perform it now, so I wonder if it is possible to collect sufficient information from literature on sensitive species cover changing in relation to environmental gradients. The authors of this paper referred to several studies dealing with this problem and some additional references are provided below.

Answer to comment number 4:

The proposed method could be easily used at different geographical scales or under different ecological settings identifying the appropriate target species in the way describe on lines 543-547. In fact, one of the proposed target species for our study area (the red gorgonia *Paramuricea clavata*) showed sensitivity for thermal anomalies in different places such as Balearic Islands (Linares et al., 2017). Nevertheless, it is important to disentangle sensitivity itself from other potential confounding factors such as population dynamics showing sharp changes in coverage through the time not related to environmental disturbances. It is the main objective of the study, i.e., demonstrate that the selected species remain stable in coverage in a long-time period.

Linares C, Ballesteros E, Verdura J, Aspillaga E, Capdevila P, Coma R et al. Efectos del cambio climático sobre la gorgonia *Paramuricea clavata* y el coralígeno asociado en el parque nacional marítimo-terrestre del archipiélago de cabrera. Proyectos de investigación en Parques Nacionales: 2012-2015.

Comment 5:

The authors sustain that environmental stability of the investigated habitat is not changed during this study, and this represents a key point for the method validation, since sensitive species cover is expected to decrease only if i) coastal disturbances and ii) climate change occur. Temperature time series have been reported from the study area, confirming the stability of the environment, but no information has been reported regarding nutrients or pollutant concentration. This information is essential because the study area lye proximal to zones under anthropic pressure (es. Bay of Algeciras) and it is subjected to a high level of marine traffic and polluting events.

Answer to comment number 5:

The species were selected for their sensitivity to chemical contamination and other sources of environmental perturbation based on other previous studies that have been properly referenced. Therefore, the relationship between absence of industrial or sewage pollution and the presence of these target species is a well established evidence in the scientific literature. As we have indicated, the objective of the study was to determine that the coverage of these species remain stable in the long term under normal environmental conditions. No significant contamination event has been reported for this period in the study area according to the available information by environmental authorities.

Comment 6:

Line 65. I think this reference could be of interest:

de Juan, S., & Demestre, M. (2012). A Trawl Disturbance Indicator to quantify large scale fishing impact on benthic ecosystems. Ecological Indicators, 18, 183-190.

Line 71. Please consider this reference:

Gobert, S., Sartoretto, S., Rico-Raimondino, V., Andral, B., Chery, A., Lejeune, P., & Boissery, P. (2009). Assessment of the ecological status of Mediterranean French coastal waters as required by the Water Framework Directive using the Posidonia oceanica Rapid Easy Index: PREI. Marine Pollution Bulletin, 58(11), 1727-1733.

Line 82-83. I agree that short-term studies are more common than long-term ones, but nowadays long-term studies are becoming not so rare. Here I report some additional examples.

Bianchi, C. N., Cocito, S., Diviacco, G., Dondi, N., Fratangeli, F., Montefalcone, M., ... & Morri, C. (2018). The park never born: Outcome of a quarter of a century of inaction on the sea‐floor integrity of a proposed but not established Marine Protected Area. Aquatic Conservation: Marine and Freshwater Ecosystems, 28(5), 1209-1228.

Betti, F., Bavestrello, G., Bo, M., Asnaghi, V., Chiantore, M., Bava, S., & Cattaneo‐Vietti, R. (2017). Over 10 years of variation in Mediterranean reef benthic communities. Marine Ecology, 38(3), e12439.

[130] Montefalcone, M., Morri, C., Bianchi, C. N., Bavestrello, G., & Piazzi, L. (2017). The two facets of species sensitivity: Stress and disturbance on coralligenous assemblages in space and time. Marine pollution bulletin, 117(1-2), 229-238.

Bertolino, M., Betti, F., Bo, M., Cattaneo-Vietti, R., Pansini, M., Romero, J., & Bavestrello, G. (2016). Changes and stability of a Mediterranean hard bottom benthic community over 25 years. Journal of the Marine Biological Association of the United Kingdom, 96(2), 341-350.

Line 104. I suggest changing with “Study area”.

Line 160-181. This paragraph reports several repetitions and inaccurate information. I suggest to delete it.

Line 161-166. This information has been already provided.

Line 166-172. Copied and pasted from lines 105-112.

Line 175-176. I suggest moving this sentence in the next paragraph.

Line 176-181. I understand that a summary of the SBPQ method could help readers in understanding the ms. Anyway, I found this part confusing. Please delete or rephrase it.

Line 203. Only one location is mentioned in lines 141-142. Please clarify this part.

Answer to comment number 6:

All suggestions have been taken into account in the revised manuscript. The proposed references have been included.

Comment 7:

Line 208. Please provide more information on the month/season selected for annual sampling.

Answer to comment number 7:

All samples were performed between the spring and summer seasons.

Comment 8:

Line 267. In order to help readers, I suggest separating the results from the discussion sections.

Answer to comment number 8:

It has been done.

Comment 9:

Line 283. In my opinion, the differences observed in Fig. 4 are significant. I understand that the RM-ANOVA does not detect significative differences, but I can clearly see that *A. calycularis* (V) cover increases from about 10-15% to 25-30% and *P.clavata* (V) cover decreases from about 30-35% to 20-25%.
Please note that if the decreasing trend showed by *P. clavata* is maintained, this species is expected to disappear in the next 20-30 years. Furthermore, a similar decreasing trend in *P. clavata* coverage over 10 years has been observed by Betti et al., 2017 and I think it should be properly considered in the discussion section.

Answer to comment number 9:

The following sentence has been included in the discussion section (line 356). "The result of the analysis shows a decrease in the coverage of *P. clavata* throughout the studied period. This circumstance coincides with that observed in other studies (Betty et al., 2017). Environmental factors such as climate change may be affecting certain species more slowly. These trends may be analyzed when they have a longer period.”

Comment 10:

Line 314. I understand the importance to demonstrate that the orientation factor deeply influences coralligenous assemblages, but this is not the main objective of this paper. This evidence is highlighted by the RM-ANOVA and RM-PERMANOVA analyses (Tables 1 and 2) and from Figures 3 and 4. Thus, I think that the nmMDS (Fig. 5) is redundant. Have authors considered any multivariate statistic method to verify that there are no significative differences among species cover in vertical quadrats?

Answer to comment number 10:

The two multivariate statistic methods used has been RM-PERMANOVA and nmMDS. The latter, unlike the first, is an indirect gradient analysis approach which produces an ordination based on a distance or dissimilarity matrix and, although it may be expendable as the reviewer suggests, we recommend its inclusion in the paper as it is a very visual graph, and serves as a reinforcement of the RM-ANOVA analytics and RM-PERMANOVA carried out. However, we are opened to include other different multivariate analysis that the referee considers appropriate to suggest.

Comment 11:

Line 320. A “Discussion” section could start here.

Answer to comment number 11:

It has been done.

Comment 12:

Line 419-420. Please see my previous comment (a).

Answer to comment number 12:

Our methodology is low cost and its implementation is simple. The ultimate goal is to create a network in which not only scientists and technicians participate, but also a diving club or volunteer groups.

Comment 13:

Line 421. “This species” is not clear in this sentence.

Answer to comment number 13:

The environmental information provided by these sessile benthic species is significant because it covers a wide range of sessile organisms that are long-lived and sensitive to changes in the environment.

Comment 14:

Line 461. Table 3. I suggest inserting an additional column with each geographical location. I also suggest to report first author name and year of publication for the studies in the first column.

Answer to comment number 14:

The table 3 has been inserted an additional column with each geographical location and first author name and year of publication for the studies has been reported in the first column.

Comment 15:

I strongly suggest to include a supplementary material table, including all the data collected during this study. Authors could use two SM tables, one for each orientation (vertical/horizontal), to report the mean values of coverage for the target species during the 10 years.

Answer to comment number 15:

A supplementary material table has been included.

Comment 16:

A total of 320 pictures were analyzed in this study. I suggest to include a new figure in the main text, showing a picture taken in 2005 and the same picture taken in 2014. This could be done for both vertical and horizontal quadrats, for a total of 4 pictures in the figure.

Answer to comment number 16:

 A new figure (figure 7) that showing the pictures requested.
