# Supplementary material for: Detect coastal disturbances and climate change effects in coralligenous community through sentinel stations
Source: PLoS One. 2020 May 5;15(5):e0231641. doi: 10.1371/journal.pone.0231641 (PMC7200002; doi:10.1371/journal.pone.0231641)
Supplement: Attachment — Submitted filename: Responses to reviewers.docx [file pone.0231641.s004.docx]

Responses to reviewers

Reviewer #2: we have incorporated your suggestions into revisión. We want to thank you for your contributions to improve the manuscript. We present your numbered comments and corresponding response.

Comment 1:

The validation of the method represents the key aspect of this work: authors state that the main goal of this paper is “to validate the Sessile Bioindicators in Permanent Quadrats (SBPQ) underwater environmental alert method” (lines 24, 120, 523). The validation of a method must be done by using totally independent dataset in which you apply the method and look at the results. Otherwise, you are using a circular argument.

Answer to comment number 1:

The study aims to confirm the degree of coverage stability for the selected species throughout the monitored period, this is essential for the method to be effective but does not mean that it has been fully validated. It is true that it is not correct to use the term "validation" in the indicated lines. The term "validation" has been changed to "assessment" throughout the manuscript.

On the other hand, indicate that the sentry stations that have detected the presence of the *Rugulopterix okamurae* algae are not in the same location as those used in the present study.

Comment 2:

The reference proposed by the authors (see the answer to my comment 2 in the previous revision) may prove that the method is able to detect the presence of invasive species, but it still does not prove that the SBPQ properly detects the several environmental changes that authors list in the text (line 174): “This methodology is a simple, non-invasive, underwater environmental alert tool for the early detection of environmental impacts of anthropic origin in the sublittoral system: in the short term (local alterations derived from pollutants from industries or emissions of urban origin, coastal dredging or civil engineering works on the coast, intrusion of exotic species with invasive potential, among other sources of alteration of coastal waters), and in the medium or long term (global warming)”.

Answer to comment number 2:

We agree with your comment. The cited reference can only demonstrate that the methodology is effective to detect invasive species but not with respect to other situations. Global warming has been included in the discussion of the manuscript because in the article commented above, it is hypothesized that this type of invasive species may be favored by climate change. Textually: a) in the text of the referred article (pag 7) : "The explosive growth of R. okamurae documented in a very short time in Ceuta also coincided with the maximum SST in the Strait of Gibraltar and nearby areas in the period between 2000 and 2017, peaking at 23.9 ºC in July 2015"; b) in the Abstract (pag 1): "This bloom could have been associated with the temperature peak in July 2015 and was thus possibly linked to global warming".

It is necessary to test the methodology regarding different environmental disturbances to assess the response of the method. Line 174 has been modified to be less categorical.

*“This methodology has been designed as a simple, non-invasive, underwater environmental alert tool for the potential early detection of environmental impacts of anthropic origin in the sublittoral system: in the short term (local alterations derived from pollutants from industries or emissions of urban origin, coastal dredging or civil engineering works on the coast, intrusion of exotic species with invasive potential, among other sources of alteration of coastal waters), and in the medium or long term (global warming). Even though the method is able to detect the presence of invasive species, further studies are required to test the reliability of the method for detecting other potential impacts of anthropic origin”*

Comment 3:

In my opinion, the text should focus on the long-term monitoring, the 10-years dataset and the method employed to obtain it, avoiding the use of “validation” or “to confirm the untested part of the method” (line 34, 126, 528).

Please note also that the description of the method itself is long and difficult to read, reporting information of scarce relevance, whereas the description of the outputs processing (which is, in my opinion, of major relevance) is still lacking (see my comment 3 in the previous revision).

Answer to comment number 3:

The use of “validation” or “to confirm the untested part of the method” has been removed in the lines indicated.

As a response to the request indicated in comment 3 in the previous revision, the method was described in detail in the previous revised manuscript. We have tried to describe all the important aspects about the design and application of the method. The references used to establish the defined coverage percentage classes also was included. We think it can be interesting that the method is described in an open access scientific journal where the proposal has been validated through a peer review process and the methodology can be easily consulted by the stakeholders. We appreciate the suggest to include a more detailed description of the SBPQ methodology within the M&M section.
